# Uterine Natural Killer Cells

**DOI:** 10.3389/fimmu.2019.00960

**Published:** 2019-05-01

**Authors:** Dorothy K. Sojka, Liping Yang, Wayne M. Yokoyama

**Affiliations:** Rheumatology Division, Washington University School of Medicine, St. Louis, MO, United States

**Keywords:** uterine natural killer cells, pregnancy, tissue-resident natural killer cells, placenta, maternal-fetal interface, uterine innate lymphoid cells, conventional natural killer cells

## Abstract

Natural killer (NK) cells are members of a rapidly expanding family of innate lymphoid cells (ILCs). While most previously studied NK cells were derived from the mouse spleen and circulate in the blood, recently others and we found tissue-resident NK (trNK) cells in many tissues that resemble group 1 ILCs (ILC1s). During pregnancy, NK cells are the most abundant lymphocytes in the uterus at the maternal-fetal interface and are involved in placental vascular remodeling. Prior studies suggested that these uterine NK (uNK) cells are mostly derived from circulating NK cells. However, the murine virgin uterus contains mostly trNK cells and it has been challenging to determine their contribution to uNK cells in pregnancy as well as other potential function(s) of uNK cells due to the dynamic microenvironment in the pregnant uterus. This review focuses on the origins and functions of the heterogeneous populations of uNK cells during the course of murine pregnancy.

## Introduction

Innate lymphoid cells (ILCs) constitute an expanding heterogeneous family of cells that are found resident in tissues (1–4). Unlike T and B lymphocytes, ILCs do not require RAG-dependent somatic rearrangement for expression of their receptors. ILCs can respond early to eliminate virally infected and transformed cells and provide epithelial barrier immunity. ILCs form complex interactions with tissue-specific cells where they integrate signals and respond appropriately to maintain tissue homeostasis and repair, expanding their functions beyond host immunity.

A recent re-classification categorized ILCs into five subsets based on transcription factors and cytokine production: ILC1s, ILC2s, ILC3s, lymphoid tissue-inducer (LTi) cells and conventional NK (cNK) cells (5, 6). TBET^+^ ILC1s produce type 1 cytokines IFN-γ, TNF-α and GM-CSF; GATA3^+^ ILC2s produce type 2 cytokines IL-5 and IL-13; and RORγT^+^ ILC3s cells produce IL-17 and IL-22. LTi cells are also RORγT^+^ and are important in formation of secondary lymphoid structures but do not produce IL-17 or IL-22. Similar to ILC1s, cNK cells produce IFN-γ but possess a much higher cytotoxic potential, differentiating them from the ILC1s. Because of their tissue occupancy, ILCs are privy to local dysregulation and pathogenic insult and collectively appear to have a diverse toolbox to not only combat infection but also restore tissue homeostasis by initiating tissue repair mechanisms (7–10). Hence, at the tissue site, the multidimensional biology of ILCs allows for a prompt response to meet the needs of the altered tissue.

ILCs are resident in many tissues throughout the body (1). Cells resembling cNK cells and ILC1s are enriched in several organs (11–15). We identified two populations of murine NK cells, tissue-resident (trNK) and circulating cNK cells that occupy non-lymphoid tissues such as the liver, skin, and virgin uterus (13, 14). The virgin uterus contains an abundant number of trNK cells and a few cNK cells, often described as negligible. The highly specialized uterine tissue, with cyclic exposure to sex hormones and invading extravillous trophoblast during pregnancy, contains trNK cells, cNK cells, and ILC1s, here referred to as uterine NK (uNK) cells to include all subsets (15, 16). In this review we provide an overview of uNK cells, with a focus on mouse.

### Conventional NK Cells

Most of our knowledge about the phenotype, function and development of murine NK cells comes from studying NK cells found circulating in the blood and spleen, here termed conventional NK (cNK) cells. The cNK cell population constitutes 2–3% of the lymphocytes in the blood and spleen where they have been extensively studied. Functionally, they are set apart from other ILCs because of their potent cytotoxic capability to potentially kill on contact. ILC developmental studies determined that all ILC lineages are derived from early common lymphoid progenitors (CLPs) that can give rise to NK cells, ILC1s, ILC2s, ILC3s, and LTi cells (17–19). The common progenitor to all helper-like innate lymphoid cell lineages (CHILP) gives rise to PLZF^+^ ILC precursors that develop into ILC1s, ILC2s, ILC3s, and LTi cells separating them from the NFIL3^+^ NK precursor that give rise to cNK cells earlier in the developmental pathway (20). Therefore, cNK cells are developmentally distinct members of the ILCs.

### Tissue-Resident NK Cells

Circulating cNK cells are widely distributed throughout the body but many tissues, have resident NK cells, termed tissue-resident NK (trNK) cells that are present in the liver, skin, kidney and virgin uterus (13, 14, 21). Although cNK and trNK cells are both absent in IL15Rα-deficient mice demonstrating they both depend on IL-15 signaling in early development, there are several characteristics that distinguish cNK and trNK cells. First, surgical joining of two congenically marked animals in parabiosis studies determined that the cNK cells traffic freely in the circulation while the trNK cells remain in the tissue (13, 14, 16). Second, detailed phenotypic and RNA-seq analyses revealed that cNK and trNK cells differentially express receptors and transcription factors that can be used for their identification. The cNK cells express the integrin DX5 but lack the expression of another integrin, CD49a, and are defined as CD3^−^NK1.1^+^CD49a^−^DX5^+^. In a mutually exclusive manner for DX5 and CD49a staining, trNK cells lack expression of DX5 but express CD49a and are defined as CD3^−^NK1.1^+^CD49a^+^DX5^−^. All cNK cells require the transcription factors *Nfil3* and Eomesodermin for development while trNK cells do not. In contrast, *Tbet*, which has a less profound effect on cNK cell development, is required for the development of trNK cells in liver and skin. Interestingly, uNK cells in the virgin uterus are predominantly trNK cells and develop independent of both *Nfil3* and *Tbet* (13), strongly suggesting that they form a lineage distinct from cNK cells and trNK cells in liver or skin. Taken together, these data indicate that cNK and trNK cells represent different lineages of NK cells rather than different differentiation states.

### ILC1s

The trNK cells and ILC1s share features but have important differences making it difficult to use the terms interchangeable to define a population. Both trNK cells and ILC1s are resident populations in tissues (1, 13, 14) and both express receptors that have been used to define NK cells such as NK1.1 and NKp46. In the case of the trNK cells in the liver, developmental studies indicate that they use the ILC1 precursor pathway distinguishing them from the cNK developmental pathway (20), making the term ILC1 an appropriate term to define the trNK cells in the liver. However, developmental studies are lacking for ILCs in uterine tissue and trNK cells in the murine virgin uterus develop independent of Tbet, which is required for all ILC1s and liver trNK cells. Therefore, caution needs to be taken when a population is solely defined phenotypically as marker expression may vary among different tissue microenvironments.

## Uterine Adaptation Throughout Gestation

Uterine adaptation to pregnancy supports fetal growth by the formation of a maternal-fetal interface. Despite structural placental differences between mouse (labyrinth) and human (villous), the uterine tissue response to pregnancy is very similar between the two hemochorial placental species (22), with the fetal chorion directly bathing in maternal blood. These pregnancy-induced responses include uterine receptivity to blastocyst implantation, endometrial decidualization, placental vascular remodeling, and maternal immune cell composition at the maternal-fetal interface. The gestational timeline is well-established during murine pregnancy and continues to be a valuable model to study pregnancy-related physiology and pathology.

**Figure 1:**
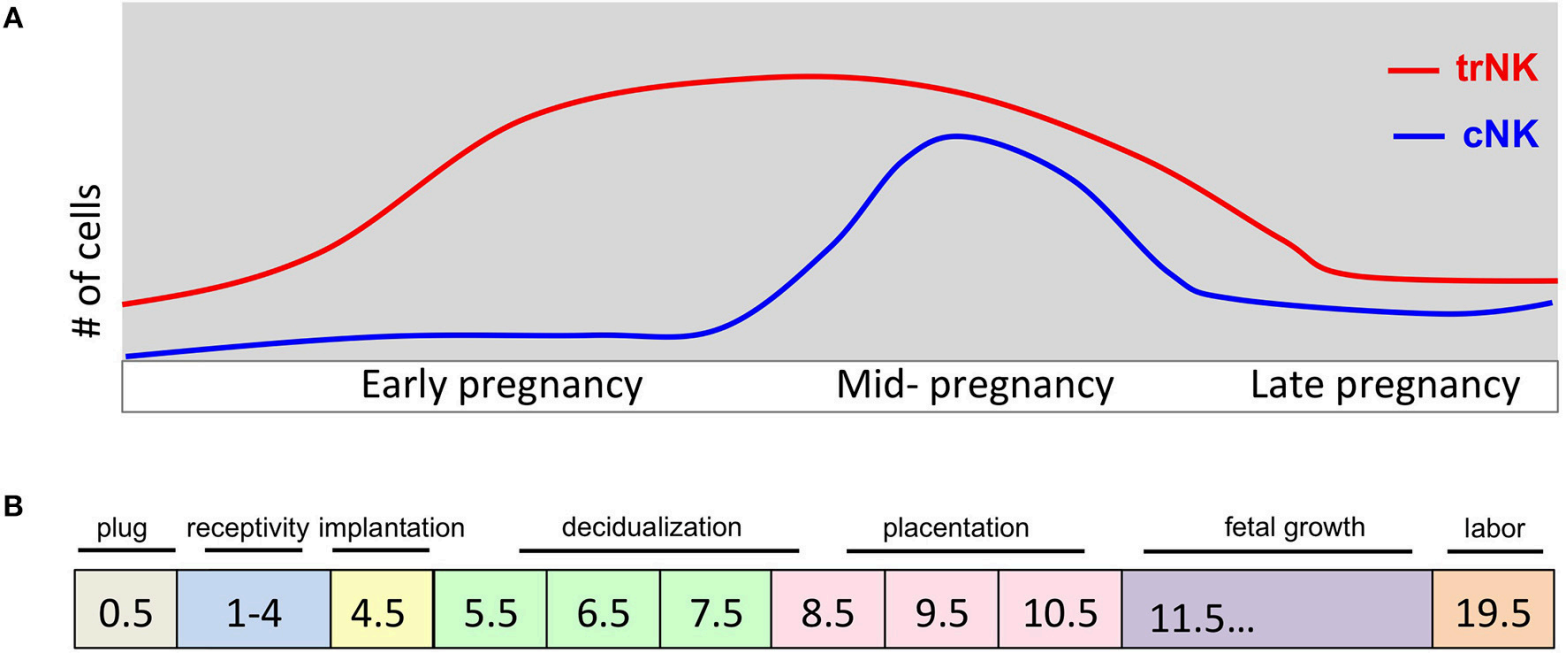
Pregnancy events and uNK cell kinetics during murine pregnancy. **(A)** Schematic diagram of number (y-axis) of trNK and cNK cells during pregnancy (gd on x-axis). During early pregnancy the trNK cells dominate the virgin and decidualized endometrium. By mid-gestation, cNK cells are increased in number and both trNK and cNK cells decline during late pregnancy. **(B)** Schematic diagram of key events during pregnancy at indicated gd's. Uterine adaptation to pregnancy begins shortly after the visualization of a copulation plug and before embryo implantation, identified as window of uterine receptivity. During this time the uterine tissue is prepared for embryo implantation. Embryo implantation triggers the process of decidualization causing extensive proliferation and vascular modification initiating the process of placentation. A fully developed placenta marks mid-gestation.

The mouse uterus undergoes dynamic changes that accompany the developing conceptus from implantation to the main event, parturition (Figure 1B). In C57BL/6J mice, the gestational length is 19.5 days (gd19.5) while in humans it is 40 weeks. When embarking on mouse pregnancy studies, investigators must be aware that specific animal facility characteristics such as food, water, bedding, noise pollution and animal husbandry can all affect gestational length. There are also mouse strain-dependent variations in gestational length so it is important to breed controls of the same genetic background when assessing transgenic models for reproductive fitness (23). One of the most accurate methods for estimating gestational length is a restricted mating period (24). This is recommended and most often done with an overnight breeding strategy in which an estrus-stage dam is placed with a stud male and checked for the presence of a copulation plug before 8:00 am the next day. This method is effective because mice are nocturnal animals and fertilization typically occurs around midnight, the halfway point of a 12 h dark/light cycle (25). If a copulation plug is visualized, the mouse is identified as at gestational day (gd) 0.5, which is important to time accurately because major changes rapidly occur during early stages of mouse pregnancy. For preterm birth studies, a more precise gestational length determination is required and a 2–4 h mating period strategy is critical to follow (24).

The copulation plug, an indication that mating occurred is most often followed by pregnancy, but not always. Following the next couple of days, the uterus needs to experience a necessary estrogen surge at gd 3.5 in order to activate the window of implantation which puts the luminal epithelium in a receptive state to bind the blastocyst at gd4.5 (26). In mice blastocyst implantation initiates the endometrium transformation process called decidualization and the vascular permeability and immune cell accumulation that are associated with the process.

Decidualization begins at gd6.5 and is characterized by extensive cell proliferation and remodeling. Fibroblast cells proliferate and differentiate into decidual cells that assume an epithelial cell-like phenotype. Extracellular matrix remodeling of the endometrial stroma and angiogenesis are initiated during decidualization and continue until the placenta is fully formed. Additionally there is a marked increase in immune cells; specifically uNK cells, beginning with the onset of decidualization (16, 27). The embryo becomes completely surrounded by the decidualized endometrium at which time the primitive placenta, called the choriovitelline placenta, is the main source of nutrition for the developing embryo between gd 6.5–10.5 (28, 29). In human pregnancy, decidualization is triggered during the menstrual cycle, independent of implantation (30). Decidualization is essential for a successful pregnancy to ensue as insufficiency in decidualization can cause infertility and recurrent spontaneous abortion.

**Figure 2:**
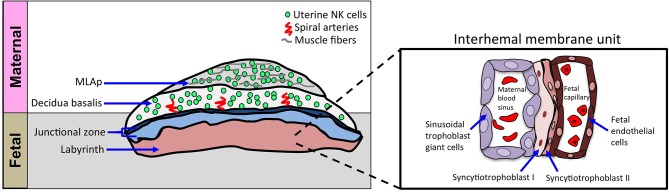
Schematic of cellular structures of mouse definitive chorioallantoic placenta. Schematic diagram of placenta, oriented with maternal tissues above fetal tissues, as indicated. The inset shows a closer view of the interhemal membrane unit in the placental labyrinth. The murine chorioallantoic placenta, at gd11.5, is fully developed. The maternal contributions to the chorioallantoic placenta are the MLAp and the decidua basalis, both regions dominated by uNK cells. The fetal-derived invading trophoblasts can be found in the decidua basalis and with the uNK cells they aid in spiral artery remodeling during placentation. The spongiotrophoblast layer and parietal trophoblast giant cells (P-TGCs) layer make up the junctional zone that separates the placenta labyrinth from the decidua basalis. The labyrinth contains a highly organized cellular barrier called an interhemal membrane unit that separates the maternal blood from the fetal blood. The maternal blood sinus is lined with sinusoidal trophoblast giant cells and separated from the fetal blood compartment by two layers of syncytiotrophoblast cells (SynT-I and SynT-II). The fetal endothelial cells line the fetal capillaries.

The murine definitive placenta, chorioallantoic placenta, is considered fully developed and assumes nourishment of the developing embryo at gd10.5–11.5 when four distinct compartments can be histologically distinguished (Figure 2). Farthest away from the fetus is the mesometrial lymphoid aggregate of pregnancy (MLAp) embedded in the myometrium of the uterine wall and specific to murine pregnancy. Underneath the MLAp is the decidua basalis, which contains immune cells, invading trophoblasts and the remodeled vasculature, and which in mouse does not extend into the MLAp. The junctional zone consists of spongio-trophoblast (SpT) and glycogen trophoblast cells (GlyT), and a layer of parietal trophoblast giant cells (P-TGCs) that provides a separation between the maternal decidua basalis and the labyrinth. Closest to the fetus is the labyrinth, the innermost compartment of the placenta. The interhemal membrane unit, also known as the exchange barrier, in the labyrinth is made up of three trophoblast cell types and an endothelial cell layer of the fetal vasculature (Figure 2 inset). Sinusoidal trophoblast giant cells line the maternal blood sinus, which is separated from the fetal blood capillary by two barrier layers, syncytiotrophoblast I and II. Moreover, the invasive extravillous trophoblast cells are intimate with the maternal immune cells and both are in the same space, the decidua basalis. Together they provide the structure of the remodeled vasculature.

The extent of trophoblast invasion differs between mouse and human, with more extensive invasion into decidua stroma, arteries and myometrium in the latter (31). In human, pregnancy complications linked to inadequate placental perfusion such as preeclampsia and intrauterine growth restriction (IUGR) are associated with inefficient extravillous trophoblast invasion (32). Hence, uterine adaptations to pregnancy establish and maintain the maternal-fetal interface.

## Maternal-fetal Interface

The development of the maternal-fetal interface is critical for the successful outcome of pregnancy. The maternal component of the interface is the decidua basalis, which contains the maternal immune cells and the fetal component is the placenta labyrinth, which contains fetal-derived invading trophoblast cells (Figure 2). The maternal-fetal interface is common ground for the two allogeneic entities to communicate. At the center are the uNK cells that have been implicated in remodeling of the placental vasculature, regulating invading trophoblast cells, and providing immunity.

## NK Heterogeneity in the Uterus

Granulated metrial gland (GMG) cells were first visualized and characterized by light microscopy over a century ago and more recently by electron microscopy. Identified by morphologists as a prominent cell population containing cytoplasmic granules and occupying the metrial gland during pregnancy, the metrial gland is induced during murine pregnancy and is embedded between the muscle layers of the uterus at the implantation site (33). Since the metrial gland was not of epithelial cells, did not resemble glands histologically, and did not have endocrine or exocrine functions, Croy proposed to rename the structure (34). As a result, two names have been used interchangeably in the literature to replace the term granulated metrial gland: mesometrial triangle and MLAp (34–36). In this review we will refer to this structure as MLAp. Concurrently with the name change, studies revealed that GMG cells belonged to the NK cell lineage and have since been referred to as uterine NK (uNK) cells, as well (37).

During murine pregnancy, uNK cells make up the vast majority of the maternal leukocytes, constituting 70% of the lymphocyte fraction (38–40). Histological analysis revealed uNK cells to occupy both the MLAp and the decidua basalis of the implantation site in early pregnancy, with a decline in both locations at parturition. Heterogeneity among the uNK cells was identified by differences in size and cytoplasmic granule content, which correlated with the maturation status, with the smaller uNK cells mostly residing in the MLAp. Classically identified by histological approaches, uNK cells were detected by the periodic acid-Schiff (PAS) reaction with or without co-reactivity by *Dolichos biflorus* agglutinin (DBA) lectin staining, with DBA reactivity specifically found in the murine pregnant uterus (41). The DBA^+^ cells are often referred to as decidual NK cells in the literature. Flow cytometry helped to further characterize uNK cells, which expressed CD45 and NK cell-specific receptors and lacked expression of T cell, B cell and macrophage markers. Analysis of *Rag2*^−/−^γ*c*^−/−^ mutant mice, deficient in cNK cells, also indicated absence of uNK cells. When reconstituted with wild-type bone marrow, they showed uNK cell development in the uterus, consistent with bone marrow derivation of uNK cells (42, 43). Taken together, the morphologic, phenotypic and bone marrow reconstitution experiments supported the assignment of uNK cells to the NK cell lineage.

**Figure 3:**
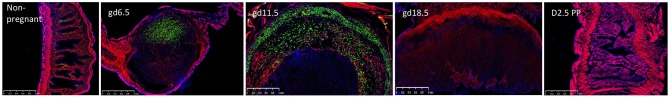
The accumulation and decline of GFP^+^ uNK cells in the Ncr1^iCre^ X Rosa^mT/mG^ reporter mice during pregnancy. The GFP^+^ uNK cells accumulate in the decidualized endometrium at gd6.5 and are found segregated to the myometrium and decidua basalis by gd11.5. At gd18.5, (laboring dam) the placenta has remnants of the MLAp and decidua basalis and is essentially devoid of uNK cells. Remarkably by 2.5 days post-partum, the structure of the uterus is remodeled and contains uNK cells. Uterine sections were derived from non-pregnant mouse, different gd's, and 2.5 days post-partum, as indicated.

Recently, we used a novel NK reporter mouse to visualize the emergence of uNK cells during pregnancy (Figure 3). Since the *Ncr1* gene encodes NKp46, a receptor selectively expressed on all NK cells, *Ncr1*^iCre^ mice restrict improved Cre (iCre) expression to NK cells (44). *Rosa*^mT/mG^ mice (45) contain a construct with membrane-bound Tomato constitutively expressed in all tissues. When Cre is expressed, the Tomato cassette and a stop codon are excised, allowing for expression of membrane-bound GFP and fate mapping of essentially all NKp46^+^ NK cells in *Ncr1*^iCre^ x *Rosa*^mT/mG^ mice. We confirmed the GFP^+^ cells in the uteri of these reporter mice were indeed NK cells based on flow cytometry phenotypic analyses with NK cell-specific markers (16). An extensive time course revealed that at gd6.5 the decidua basalis contained proliferating GFP^+^ uNK cells, prior to the development of MLAp, challenging the proposed idea that the MLAp was a source of immature uNK cells (46). At gd 10.5 (mid-gestation), we found a prominent MLAp structure and a fully developed decidua basalis, both of which contained GFP^+^ uNK cells, unlike the placenta labyrinth (16). Shortly after mid-gestation, the GFP^+^ uNK cells began to decline in number to essentially non-existent at the implantation site in a laboring dam. Remarkably, at 2.5 days post-partum, GFP^+^ uNK cells start to resemble those in the non-pregnant uterus (Figure 3). Hence, the *Ncr1*^iCre^ x *Rosa*^mT/mG^ mice allow detection of GFP^+^ uNK cells with greater sensitivity, particularly with easily detectable GFP^+^ uNK cells in the MLAp (16) that can be further analyzed by histological analysis and by flow cytometry.

## Origin of uNK Cells

The origin of uNK cells during pregnancy has been of long standing interest. Whether mouse uNK cells in the pregnant uterus develop *in situ* from progenitor cells in the virgin uterus or home there from the periphery had been addressed using several approaches that include uterine segment transplantation, adoptive transfers and parabiosis. Here we will summarize these studies before describing more recent studies that provide a new hypothesis.

Previously, uNK cell origin was studied by uterine segment transplantation (47, 48). Wild-type (WT) uterine segments from virgin mice were engrafted into the uterine horns of either NK-deficient (*Rag2*^−/−^ γ*c*^−/−^) or NK-sufficient (WT) controls. After the uterine segments established end-to-end anastomosis, the mice were mated and analyzed histologically on gd10. Uterine segments transplanted into WT hosts contained uNK cells but when transplanted into NK-deficient hosts, no uNK cells were found despite having a decidualized uterus originating from WT (NK-sufficient) donors. These data demonstrated that peripheral NK cells homed to the uterus and that the uterus did not contain uNK cells that expanded during pregnancy, with the caveat of possible surgical effects on the host uterine tissue. Regardless, in another approach, adoptive transfer of bone marrow, thymus, lymph node, and spleen or fetal liver cells from SCID mice into alymphoid recipients resulted in detection of donor-derived uNK cells in the pregnant uterus (43), providing further support for NK or progenitor cell homing. This homing to the uterus was independent of chemokine receptors CCR-2 and CCR-5 (49) but specific chemokine receptors have not been identified. However, a recent study disputed these findings as transferred splenic NK cells from *virgin* mice did not home to the pregnant uterus and already present uNK cells appeared to expand (50).

Previously, we reported that murine virgin uteri contain few circulating CD49a^−^ DX5^+^ cNK cells and an abundant CD49a^+^ DX5^−^ trNK cell population (13). A subset of the CD49a^+^ DX5^−^ trNK cell population in the uterus was found to lack the expression of Eomesodermin and identified as ILC1, with trNK cells still dominating the uterus during early pregnancy (15, 16, 27, 51, 52). The accumulation of uNK cells during pregnancy could be due to local proliferation of trNK cells and ILC1s, migration of cNK cells or a combination of both. In the decidua basalis during early pregnancy (gd6.5), trNK cells but not cNK cells were highly proliferative as marked by increased BrdU incorporation and high Ki67 expression (16, 27), with undetectable ILC1s in these studies. Parabiosis experiments with experimentally induced decidualization confirmed that there is minimal contribution from migrating cNK cells to the local proliferating pool of trNK cells in a model of early pregnancy (16). Taken together, these findings indicate that accumulation of uNK cells in *early* pregnancy originates from local proliferating trNK cells.

Our data do not exclude the contribution of cNK cells migrating from the periphery. Although we did not detect any indication of their proliferation, cNK cells increased in number. As previously reported, migration into the pregnant uterus could be one mechanism to account for the increase in cell number in the absence of proliferation. Taken together, we propose a new hypothesis to account for the cNK cell and trNK cell contributions to the pool of uNK cells during murine pregnancy.

We proposed a two-wave hypothesis for uNK cell accumulation in the pregnant uterus that is driven by uterine tissue remodeling events during pregnancy and takes into account uNK cell heterogeneity (Figure 1A) (16, 27). The first wave is initiated at the onset of the decidualization process where our parabiosis experiments demonstrated the local proliferation of trNK cells with minimal contribution from the circulating cNK cells (16). The second wave involves the recruitment of cNK cells during the placentation process that includes vascular remodeling. Mice that lack cNK cells but retain trNK cells, such as the *Nfil3*^−/−^ mice, have a major defect in uNK cell accumulation and placentation is suboptimal with aberrant spiral artery remodeling (53, 54). Taken together, these data support the contribution of both trNK cells and peripheral cNK cells to the uNK cell population during pregnancy.

## cNK Cell Function

NK cells can eliminate tumor cells upon contact without prior sensitization, an event known as natural cytotoxicity (55). This is in contrast to T cell-mediated cytotoxicity that requires major histocompatibility complex (MHC)-dependent antigen recognition. NK cells can also use their cytotoxic machinery and cytokine production to elicit anti-viral immunity early during an infection.

Direct contact with the target cells may engage receptors expressed on NK cells. NK cell receptors are stochastically expressed and an individual NK cell can express several different inhibitory and activation receptors simultaneously, resulting in the potential for many specificities. The NK cell receptor repertoire is dependent on the inherited haplotype of NK cell receptor genes. In the mouse, the genes for Ly49, CD94, NKG2, NKG2D, and NK1.1 (encoded by *Nkrp1*) receptors reside in the NK gene complex (NKC) on mouse chromosome 6 (56).

NK cells recognize their cellular targets via two functional types of surface receptors: activation and inhibitory (55). NK cell inhibitory receptors that engage target MHC-I and deliver negative signals via cytoplasmic immunoreceptor Tyr-based inhibitory motifs (ITIMs) that recruit Tyr phosphatase, SHP1, provide an explanation for missing-self recognition. This mechanism of activation receptor suppression holds true for the inhibitory receptors in mouse and human, lectin-like Ly49s and killer immunoglobulin (Ig)-like receptors (KIRs), respectively, that are functional orthologs. In contrast, ligand binding activation receptor chains couple to immunoreceptor Tyr-based activation motif (ITAM)-containing molecules, CD3ζ, FcεRIγ, or DAP12, that stabilize expression and transmit downstream intracellular signals resembling events found in TCR signaling. Thus, during effector responses, NK cell triggering by its cellular targets is typically dependent on integrating signals from activation and inhibitory receptors.

In the spleens of C57BL/6 mice, cNK cells express NK1.1, NKp46, and Ly49 receptors. Although the Ly49 receptors are also expressed by some trNK cells, their expression differ depending on the tissue from which they are examined. For example, ILC1s in the liver do not express the activation receptors Ly49D and Ly49H and have variable expression of the inhibitory Ly49 receptors (13). The inhibitory receptor Ly49I is differentially expressed on uterine trNK cells and is dependent on the location with no expression in the MLAp while in the decidua basalis expression is similar to that found on cNK cells (16). Hence, it is plausible that during pregnancy uNK cells may respond to their cellular targets using strategies, similar to but distinct, from those used by cNK cells.

Pregnant women with a specific KIR haplotype and fetal HLA-C genotype combination have a significantly higher risk of preeclampsia (57). Similar findings were reported in a cohort of African women, which have more genetically diverse KIR haplotypes and HLA alleles (58), strengthening the interpretation that inhibitory receptors on uNK cells interact with their fetal MHC-I ligands leading to increased susceptibility to preeclampsia. Conversely, genetic association studies indicate that a KIR activation receptor recognizing a fetal HLA ligand protects from preeclampsia (58, 59). Thus, these data suggest uNK cells respond to fetal MHC-I via their inhibitory and activation receptors to control proper placental vascularization and development.

## uNK Cell Function During Pregnancy

### Placental Vascular Remodeling

Although uNK cells were thought to belong to the NK cell lineage and contained large cytoplasmic granules, when isolated from the murine pregnant uterus, they possessed essentially no cytotoxic ability to kill prototypic NK cell-sensitive target cells (60–62). This was puzzling because NK cells are defined by their natural ability to kill targets. But their abundance in the pregnant uterus left many to wonder about their function. Since uNK cells have been visualized by microscopy from the very beginning of their discovery, they often were noted to be in close association with trophoblast cells lining the blood vessels. Pioneering work by Croy and colleagues proposed the hypothesis that during murine pregnancy uNK cells modulate placental vascular remodeling.

During pregnancy, spiral arterioles are transformed into high-capacitance, low-resistance, thin-walled vessels with large lumens (63). This vascular adaptation is thought to keep up with the nutritional demands of the growing fetus. Studies of mouse uNK cells support their role in this remodeling. Mice lacking cNK cells have defects in spiral artery remodeling during placentation that were rescued when IFNγ was injected systemically (53, 64–68). In bone marrow (BM) chimeric experiments, the remodeling defects were rescued when BM from NK-sufficient mice, but not BM from IFNγ^−/−^mice was used. Also, BM from IFNγ receptor-deficient mice was able to rescue, indicating that NK cells did not respond to IFNγ in order to rescue. Thus, IFNγ produced by NK cells contributes to spiral arteriole remodeling by acting on non-NK cells such as endothelial cells and decidual stromal cells but the exact signaling pathway to initiate their cytokine production and other aspects of uNK cell-dependent remodeling have not been elucidated.

### Growth Promoting Factors

Recently, uNK cells have been reported to directly stimulate fetal growth by producing growth-promoting factors essential for embryo development prior to the establishment of the placenta (52). The trNK cells specifically produced the growth factors pleiotrophin, osteoglycin, and osteopontin. A decrease in trNK cells secreting these growth factors in the *Nfil3*^−/^^−^ and aged mice impacted offspring from these dams, which had fetal growth-restricted pups with defects in bone development. The fetal growth deficiency and bone development were restored when the dams were reconstituted with *in vitro* expanded trNK cells that produced sufficient amounts of growth factors. Hence, this study sheds light on additional novel functional roles of trNK cells during early embryo development in pregnancy.

### Memory of Pregnancy

In human, first time pregnancies are at a higher risk for miscarriages and preeclampsia, a multifactorial disease characterized by impaired placental perfusion (69). The percentage of preeclampsia is greater among women that are pregnant for the first time when compared to women with repeated pregnancies. Likewise the uterus and placenta differ during a second pregnancy with regard to placental vascularization and trophoblast invasion (70). The following studies suggest that uNK cells may provide memory to aid in vascular remodeling of the placenta during subsequent pregnancies.

Mandelboim et al. identified a unique subset of human NK cells that only exist in repeated pregnancies (71). They defined these cells as pregnancy-trained decidual NK (PTdNK) cells. The PTdNK cells have a unique transcriptome and epigenetic signature and express NKG2C and LILRB1. When stimulated, the PTdNK cells produced more IFNγ and VEGFα, both important in vascular modification of the placenta in mouse studies (64, 67). In another recent study, single cell RNA-seq analysis of human first trimester decidua identified three uNK cell subsets termed dNK1, dNK2, and dNK3 (72) that all co-expressed CD49a, the receptor used to identify murine trNK cells in the uterus (13). The dNK1 cell subset expressed higher levels of KIRs and *LILRB1* receptors that bind HLA-C and HLA-G molecules, respectively, expressed on extravillous trophoblast. Thus, these studies propose that a previously primed uNK cell subset during the first pregnancy may function to recall subsequent pregnancies and be better equipped to support placental vascular development.

Colucci et al. tracked the emergence and decline of the ILC family of cells during murine pregnancy (51). The trNK cells are most abundant during early pregnancy while the cNK cells peak during placentation. The ILC1 population is dominant before puberty and is essentially not detected again until the second pregnancy, where it is the most abundant population. The ILC1 cells express CXCR6 and phenotypically resemble liver NK memory cells described in the contact hypersensitivity model (73). Taken together, these data provide the intriguing idea where an uNK cell subset provides a protective memory response in subsequent pregnancies and is conserved between mice and humans.

## Conclusions

In both mouse and human, uNK cells are the most prominent immune cells that occupy the maternal-fetal interface. The uNK cells appear to engage and establish complex interactions with the surrounding tissue, which impact their function. As more cell subsets are identified within the heterogeneous uNK cell population, it is anticipated that their functional heterogeneity will extend beyond vascular modification, growth-promotion and memory generation.

## Author Contributions

DS wrote the manuscript. LY provided the micrographs and WY edited the manuscript.

### Conflict of Interest Statement

The authors declare that the research was conducted in the absence of any commercial or financial relationships that could be construed as a potential conflict of interest.
